# Top 50 Most-Cited Articles on Tension Band Wiring for Patellar Fractures: A Bibliometric Analysis and Methodological Quality Assessment

**DOI:** 10.7759/cureus.97167

**Published:** 2025-11-18

**Authors:** Ahmed Zainy, Chi Hoi Lee, Hannah Sadik, Guandayi Qiao

**Affiliations:** 1 Trauma and Orthopaedics, London North West University Healthcare NHS Trust, London, GBR; 2 Trauma and Orthopaedics, Stepping Hill Hospital, Stockport, GBR; 3 Trauma and Orthopaedics, Manchester University NHS Foundation Trust, Manchester, GBR

**Keywords:** bibliometric analysis, citation analysis, methodological quality, patellar fractures, tension band wiring

## Abstract

Tension band wiring (TBW) remains the most common operation for displaced transverse patellar fractures, yet the methodological quality of highly cited papers is often questioned. We aimed to identify the 50 most-cited TBW patella papers and appraise their study designs and methodological rigour. We searched Web of Science (1990-2024) using “patella fracture AND tension band”; olecranon studies were excluded. The top 50 cited papers were selected. For each paper, we recorded bibliographic information and total citations.

Study type was assigned based on the primary topic. We scored methodological quality using the Modified Coleman Methodology Score (MCMS; 0-100; poor <55, fair 55-69, good 70-84, excellent ≥85) for clinical outcomes studies, the Methodological Index for Non-Randomized Studies (MINORS) (0-16 non-comparative/0-24 comparative) for non-randomised clinical studies, and the Biomechanics Objective Basic Quality Assessment Tool (BOBQAT) (0-100; higher scores indicate better quality) for biomechanical studies. Two reviewers scored independently, with consensus resolution. Associations between citation metrics and quality were tested using correlation and regression analyses, while subgroup comparisons were conducted using non-parametric tests to account for data distribution. Across 50 papers, the mean number of citations was 71.9 (range 29-229). Publications clustered in the late 1990s-2010s; 50% originated from the United States. Study types were biomechanical 25/50 (50%), clinical 20/50 (40%), and mixed/review/technique 5/50 (10%). Only 2/50 (4%) were small single-centre randomised trials; most clinical studies were Level III-IV. Methodological quality was moderate: MCMS mean 49.0 (SD 10.9; n=18); MINORS mean 8.8/24 (SD 2.5; n=15); BOBQAT mean 44.4/100 (SD 21.6; n=25). Citations did not show a significant correlation with methodological quality (Spearman’s ρ indicates correlation strength and direction; MCMS ρ=-0.35, p=0.153; MINORS ρ=-0.08, p=0.783; BOBQAT ρ=-0.03, p=0.879).

Among the 50 most-cited TBW patella papers, methodological quality is moderate, and citation counts do not track study rigour. The literature is dominated by biomechanical studies and lower-level clinical evidence, with very few randomised trials. Future work should prioritise prospective comparative studies with sufficient sample sizes and statistical power, including multicentre trials, to ensure that influential studies are supported by high-quality clinical evidence.

## Introduction and background

Patellar fractures play a key role in the extensor mechanism but account for approximately 1% of all bone fractures. These fractures can significantly impair knee function [1]. Tension band wiring (TBW) is a technique used to fix such fractures, particularly in the case of a displaced transverse fracture. In fact, TBW remains the single most common surgical treatment for such patellar fractures [2]. Kirschner wires (K-wires) and a figure-of-eight wire loop are used to convert the quadriceps’ tensile forces into compression across the fracture [3].

Despite its longstanding application, TBW is not without its shortcomings, which are well-known to those who have treated patellar fractures. Patients often report experiencing pain or a “pinching” sensation after the procedure. Wires may shift or break, and it’s fairly common for the hardware to be removed after the fracture has healed [1,4]. TBW also struggles with certain fracture patterns; if the patella fracture is comminuted or just a small fragment is broken off one pole of the patella, the standard tension band construct may not provide sufficient stability [1,2]. New fixation methods (such as special patellar plates or high-strength sutures) have promised improved stability and less irritation [5-8]. Despite this, there is a lack of well-supported, high-quality comparative trials guiding us on the best treatment approaches [2,9].

Nevertheless, frequently cited papers in orthopaedics, including those on TBW, may not always represent the highest level of evidence [10]. Classic studies are cited not for their methodological rigour, but for introducing a technique or principle, even if limited to small case series. Credible new studies don’t always get cited quickly; some never do. The disconnection between citation counts and real evidence quality is interesting; however, it is not reliable when examining the literature. It leads to the question that prompted this study: What are the 50 most cited papers on TBW for patellar fractures, and how robust is their methodological quality? To explore this hypothesis, we conducted a bibliometric analysis by examining citation numbers and study designs to evaluate their methodological rigour. We hypothesised (based on anecdotal experience and trends in other areas of orthopaedic literature) that many highly cited patella TBW papers might not be methodologically stellar, and that citation counts may not accurately reflect the quality of the studies. In other words, perhaps the famous papers in this niche are not the best papers, a hypothesis that has been borne out in other orthopaedic bibliometric studies [10].

## Review

Methods

Search Strategy and Eligibility Criteria

**Figure 1:**
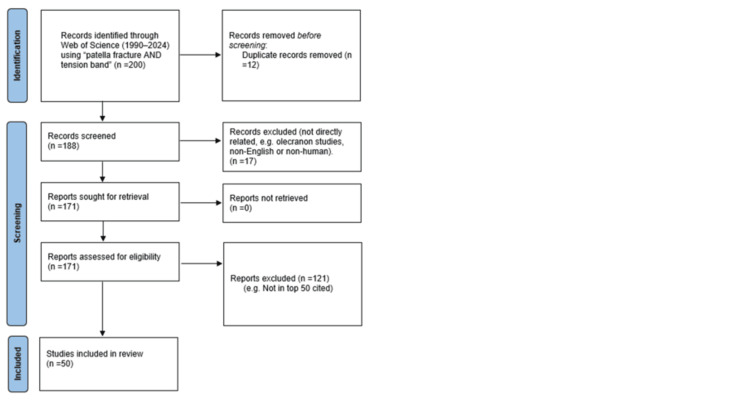
PRISMA 2020 flow diagram for identification of the 50 most-cited TBW patella fracture papers (Web of Science, 1990-2024). PRISMA: Preferred Reporting Items for Systematic Reviews and Meta-Analyses; TBW: tension band wiring

A comprehensive literature search was undertaken to identify the top 50 most cited studies on the topic of TBW for patellar fractures. To cast a wide net, we used the Web of Science database with search terms (patella fractures and tension band), restricting the results to human studies in the English language. We excluded olecranon fractures from our search to maintain focus on patellar fractures. We decided on the period from 1990 to 2024, reasoning that earlier fundamental works would be captured through citations in more recent studies. The search was conducted in early 2025, and results were sorted by citation count; the 50 papers with the highest number of citations were selected for analysis. The Preferred Reporting Items for Systematic Reviews and Meta-Analyses (PRISMA) 2020 flow diagram of identification, screening, and selection is shown in Figure 1.

Data Extraction

For each of these 50 studies, we collected basic bibliographic information: authors, year of publication, journal, and total citation count as reported by the database, as shown in Table 1. Additionally, we categorised the type of each study, which turned out to be a bit of a mix: many were clinical studies (e.g., case series of patients treated with TBW or comparative studies of TBW vs. another method), and some were biomechanical laboratory studies (testing patella constructs on the bench). The analysis highlighted the difficulty in determining how to label a study that had multiple facets (perhaps a clinical study that also conducted some biomechanical tests).

**Table 1:** Top 50 cited papers in tension band wiring of patella fractures.

First Author	Title	Citations
J.E. Carpenter [11]	Biomechanical evaluation of current patella fracture fixation techniques	229
S.T. Smith [12]	Early complications in the operative treatment of patella fractures	227
J.S. Melvin [13]	Patellar fractures in adults	197
C.T. LeBrun [14]	Functional outcomes after operatively treated patella fractures	176
C.M. Hoshino [15]	Complications following tension-band fixation of patellar fractures with cannulated screws compared with Kirschner wires	151
V.R. Patel [16]	Fixation of patella fractures with braided polyester suture: a biomechanical study	121
E.E. Berg [17]	Open reduction internal fixation of displaced transverse patella fractures with figure-eight wiring through parallel cannulated compression screws	118
C. Gwinner [18]	Current concepts review: fractures of the patella	112
C.J. Dy [19]	Meta-analysis of re-operation, nonunion, and infection after open reduction and internal fixation of patella fractures	104
S. Thelen [20]	Biomechanical cadaver testing of a fixed-angle plate in comparison to tension wiring and screw fixation in transverse patella fractures	103
J.G. Burvant [21]	Evaluation of methods of internal-fixation of transverse patella fractures - a biomechanical study	91
R. Kakazu [22]	Surgical management of patellar fractures	89
Y. Tian [23]	Cannulated screw and cable are superior to modified tension band in the treatment of transverse patella fractures	88
P.B. Wright [24]	FiberWire is superior in strength to stainless steel wire for tension band fixation of transverse patellar fractures	83
C.C. Wu [25]	Patellar tension band wiring: a revised technique	76
S.M. Chang [26]	Open reduction and internal fixation of displaced patella inferior pole fractures with anterior tension band wiring through cannulated screws	72
D.J. Schuett [27]	Current treatment strategies for patella fractures	71
M. Galla [28]	Patella fractures	65
S.C.A. Hughes [29]	A new and effective tension-band braided polyester suture technique for transverse patellar fracture fixation	65
M. Wild [30]	Fixed-angle plate osteosynthesis of the patella - an alternative to tension wiring?	64
A.P. Fortis [31]	Experimental investigation of the tension band in fractures of the patella	63
S. Thelen [32]	Cyclic long-term loading of a bilateral fixed-angle plate in comparison with tension band wiring with K-wires or cannulated screws in transverse patella fractures	62
J. John [33]	Tension-band wiring of transverse fractures of patella. The effect of site of wire twists and orientation of stainless steel wire loop: a biomechanical investigation	60
B. Schnabel [34]	Biomechanical comparison of a new staple technique with tension band wiring for transverse patella fractures	56
L. Camarda [35]	Non-metallic implant for patellar fracture fixation: a systematic review	55
J.L. Henrichsen [36]	Treatment of patella fractures	55
T. Lin [37]	Comparison of the outcomes of cannulated screws vs. modified tension band wiring fixation techniques in the management of mildly displaced patellar fractures	54
N.F. Mao [38]	Comparison of the cable pin system with conventional open surgery for transverse patella fractures	54
J. Dargel [39]	Biomechanical comparison of tension band- and interfragmentary screw fixation with a new implant in transverse patella fractures	52
S. Steinmetz [1]	Practical guidelines for the treatment of patellar fractures in adults	50
M.H.G. Heusinkveld [40]	Treatment of transverse patellar fractures: a comparison between metallic and non-metallic implants	43
K.E. Banks [41]	An alternative patellar fracture fixation: a biomechanical study	42
T.A. Scilaris [42]	Biomechanical comparison of fixation methods in transverse patella fractures	42
M. Wild [43]	Fractures of the patella	42
A.M. Chen [44]	Comparison of biodegradable and metallic tension-band fixation for patella fractures - 38 patients followed for 2 years	42
A.J. Dickens [45]	Titanium mesh as a low-profile alternative for tension-band augmentation in patella fracture fixation: A biomechanical study	40
M. Ling [46]	Where should Kirschner wires be placed when fixing patella fracture with modified tension-band wiring? A finite element analysis	40
R.M. Harrell [47]	Comparison of the mechanical properties of different tension band materials and suture techniques	40
O. Baran [48]	Anatomical and biomechanical evaluation of the tension band technique in patellar fractures	40
H.S. Neumann [49]	Long-term results after operative treatment of patellar fractures	37
B. Matthews [50]	Comminuted patella fracture in elderly patients: a systematic review and case report	37
T. Yotsumoto [51]	Tension band fixation for treatment of patellar fracture: novel technique using a braided polyblend sutures and ring pins	34
G.K.H. Shea [52]	Comparing 3 different techniques of patella fracture fixation and their complications	33
C.C. Chiang [53]	Comparison of a minimally invasive technique with open tension band wiring for displaced transverse patellar fractures	33
S.T. Nathan [54]	The management of nonunion and delayed union of patella fractures: a systematic review of the literature	33
K.L. Hsu [55]	Factors affecting the outcomes of modified tension band wiring techniques in transverse patellar fractures	32
K.A. Lefaivre [56]	Modified tension band technique for patella fractures	32
A. Rathi [57]	Percutaneous tension band wiring for patellar fractures	30
H.L. Tan [58]	Clinical results of treatment using a modified K-wire tension band versus a cannulated screw tension band in transverse patella fractures: a strobe-compliant retrospective observational study	30
Y.W. Zhang [9]	Efficacy of K-wire tension band fixation compared with other alternatives for patella fractures: a meta-analysis	29

Synthesis of Results

The method used was to assign the category based on the study's primary focus. To gauge methodological quality, we used established scoring systems to evaluate each paper. For clinical outcome studies (especially comparative or observational studies on patients), we used the Modified Coleman Methodology Score (MCMS; 0-100; poor <55, fair 55-69, good 70-84, excellent ≥85) [59]. If applicable, the Methodological Index for Non-Randomized Studies (MINORS; 0-16 non-comparative/0-24 comparative) was used for non-randomised studies [60]. For randomised trials (if any among the top 50), their Jadad score or CONSORT adherence was qualitatively noted, although there were few randomised controlled trials (RCTs) among the 50 papers. Biomechanical papers were evaluated with the Biomechanics Objective Basic Quality Assessment Tool (BOBQAT; 0-100; higher scores indicate better quality) [61]. Each scoring system was applied only to studies appropriate for that tool (i.e., MCMS and MINORS for clinical papers, BOBQAT for biomechanical), which explains why the sample sizes differ across analyses. Two authors independently scored each study, and we discussed any scoring discrepancies to reach a consensus. A separate risk of bias assessment was not performed because standard tools (e.g., the revised Cochrane Risk of Bias tool for randomised trials (RoB 2) and the Risk Of Bias In Non-randomized Studies-of Interventions tool (ROBINS-I)) are not validated for biomechanical, technique, or mixed-design studies. Instead, methodological rigour was evaluated using the MCMS, MINORS, and BOBQAT instruments, which provide structured, domain-specific quality assessment for this literature type.

Finally, we conducted simple analyses to evaluate correlations between citation counts and quality scores. This included calculating Spearman’s rank correlation coefficient (ρ) or Pearson’s correlation coefficient (r), as appropriate, and using linear regression to test whether a highly cited paper tended also to have a high methodology score, or if perhaps there was no relationship at all [10]. We also compared citation metrics between study subgroups (e.g., clinical vs. biomechanical) using non-parametric tests, since our sample size was small (50) and citation data tend to be abnormally distributed, warranting this approach (a few papers had high citation counts, acting as outliers).

Statistical Analysis

Descriptive statistics were used to summarise citation counts and methodological quality scores. Correlation between citation count and quality indices (MCMS, MINORS, BOBQAT) was assessed using Spearman’s rank correlation coefficient (ρ), given the non-normal distribution of data. For exploratory purposes, a random-effects meta-analytic model was used to pool mean methodological scores where applicable, acknowledging between-study heterogeneity. Weighting was based on inverse-variance methods. Heterogeneity was quantified using the I^2^ statistic, with thresholds of 25%, 50%, and 75% indicating low, moderate, and high heterogeneity, respectively. For all tests, a two-tailed P < 0.05 was considered statistically significant. Sensitivity analyses were performed by repeating correlation analyses after exclusion of statistical outliers. Subgroup analyses were also conducted comparing clinical versus biomechanical studies using non-parametric tests (Mann-Whitney U or Kruskal-Wallis, where appropriate). Analyses were performed using IBM SPSS Statistics for Windows, Version 29 (Released 2022; IBM Corp., Armonk, New York, United States) and R (meta package) (R Foundation for Statistical Computing, Vienna, Austria).

Results

When analysing the list of the top 50 cited papers on patella fracture TBW, the results included both expected patterns and a few notable surprises. Collectively, these 50 studies amassed an average of approximately 71.9 citations each over their lifetimes. The range was broad; the lower end had just below 30 citations, while the top paper had over 200 citations. The most individually cited article on our list accumulated 229 citations. This study was an older biomechanical study from 1997 that evaluated the mechanical fixation techniques of patella fractures [11].

**Figure 2:**
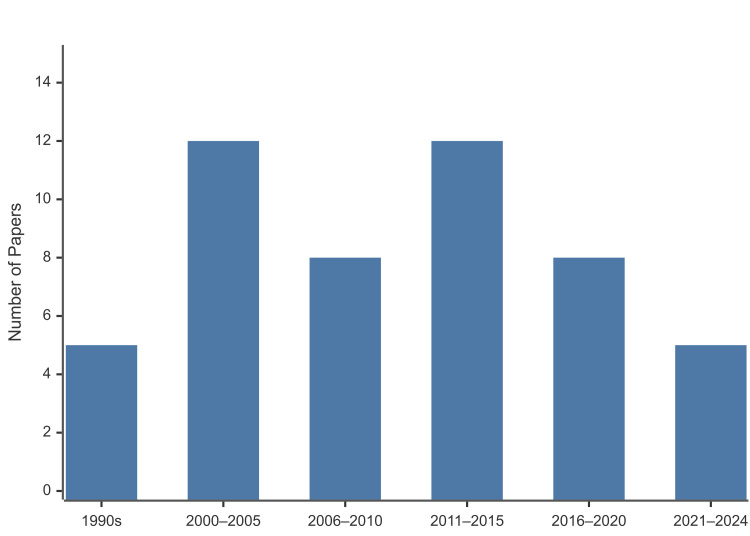
Publication periods of the 50 most-cited TBW patella fracture papers. Bars: 1990s n=5; 2000-2005 n=12; 2006-2010 n=8; 2011-2015 n=12; 2016-2020 n=8; 2021-2024 n=5. TBW: tension band wiring

**Figure 3:**
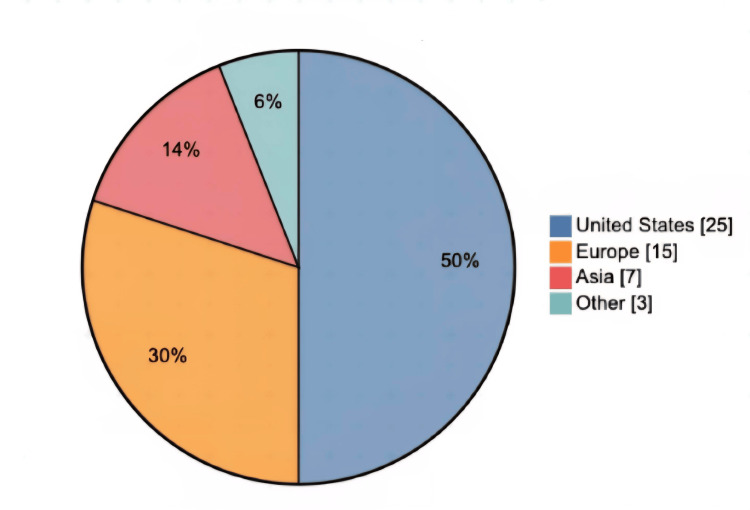
Geographical distribution of the 50 most-cited TBW patella fracture papers. United States n=25 (50%); Europe n=15 (30%); Asia n=7 (14%); Other n=3 (6%). n = 50. TBW: tension band wiring

A large number of these highly cited papers were published in the late 1990s and 2000s, with a scattering from the 2010s. This suggests that although the tension band technique originated in earlier decades, more recent papers have remained influential, likely due to increased study sizes and evolving surgical techniques. Thirty-three of the top 50 studies were published between 2000 and 2015, indicating a particularly fertile period for patellar fracture research. Publication-period frequencies are shown in Figure 2. Geographically, the majority of the studies originated from institutions in the United States, followed by contributions from Europe and Asia. In fact, similar to trends seen in other bibliometric analyses [10], roughly half of these top-cited articles came out of the US, and only a handful from any single other country. This could reflect the high research output and visibility of US orthopaedic research, or perhaps an English language citation bias, or likely a combination of factors. Geographical distribution is shown in Figure 3.

In terms of journals these studies were published in, no single journal dominated, but a few patterns emerged. Several high-impact general orthopaedic journals published the papers; the Journal of Bone and Joint Surgery (JBJS) and Clinical Orthopaedics and Related Research (CORR) each hosted a couple of our top 50. Trauma-focused journals such as Injury and Journal of Orthopaedic Trauma were also well-represented (not surprising, given patellar fractures are often considered a subset of orthopaedic trauma). We also saw contributions in sports medicine journals like The American Journal of Sports Medicine, which may seem odd for a fracture topic, but some research overlapped with sports knee injuries or relevant biomechanical aspects. Overall, the diversity of the journals reflects the interdisciplinary nature of research on patellar fracture fixation, encompassing contributions from trauma surgeons, sports medicine specialists, and general orthopedists alike.

Study Design Distribution

Among the 50 most-cited papers, approximately two-thirds were clinical studies involving patients, either retrospective case series or comparative cohorts, with a couple of small randomised trials. Around one-quarter were biomechanical laboratory studies, with the remainder consisting of review articles or technique descriptions that had been widely cited. Notably, none were large multicentre randomised trials; in fact, only two studies in our list were RCTs, and they were relatively small single-centre efforts. This indicates that the overall evidence level is likely on the lower side. The majority of clinical studies were Level IV evidence (case series or case-control studies), with some Level III (retrospective comparative studies). The analysis indicated a handful of Level II studies (prospective comparative, or lesser-quality RCTs), but certainly no Level I mega-trials within this top-cited cohort. It is interesting, albeit not surprising, that even in 2025, our hall of fame papers for patella TBW are mostly of lower evidence. This aligns with a broader trend in orthopaedic literature: influential papers tend to be the ones that pioneered a technique or filled a knowledge gap at the time, rather than being methodologically rigorous trials [10,3,62].

One illustrative example is a 2013 JBJS study by Hoshino et al., which retrospectively compared the outcomes of tension-band fixation using cannulated screws versus traditional K-wires in a large single-institution series. There was a non-significant trend towards higher fixation failure with screws versus K-wires, while elective implant removal was significantly more common after K-wire constructs; infection rates were similar [14]. Studies like that one carry weight because they address a practical question (which hardware is better?) and, even if they were not randomised trials, they provide useful data. It is easy to see why such a paper would be cited often by others debating the merits of screws versus wires.

Another cluster of top-cited papers focused on modifications to the standard TBW technique to improve outcomes. For instance, a widely referenced biomechanical study in 2009 tested a FiberWire (strong braided suture) tension band in cadaveric patella fractures and found it to be stronger than the traditional stainless steel wire configuration [6]. That finding, “FiberWire is superior in strength to stainless steel wire for tension band fixation,” has been extensively quoted in numerous subsequent papers as a rationale for trying suture-based or hybrid fixation methods. Biomechanical papers like this, which introduce an innovation or a performance improvement, tend to rack up citations quickly; this is especially true for those published in easily accessible journals. Yet, such biomechanical data do not directly predict clinical outcomes; they answer one piece of the puzzle (strength, in this case) but not the whole picture. This may partly explain why citation count doesn’t align with overall study quality or level of evidence. Researchers cite lab results because they fill practical gaps or introduce novel concepts, both of which can be highly influential in shaping surgical practice.

When assessing methodological quality, the picture was moderate-to-low. For clinical studies (MCMS, 0-100), the mean was 49.0 (SD 10.9; n=18). For non-randomised clinical studies (MINORS), normalised to a 24-point scale, the mean was 8.8/24 (SD 2.5; n=15). Many studies were retrospective, and most lacked robust controls. Biomechanical studies (BOBQAT, 0-100) did not score highly overall: mean 44.4 (SD 21.6; n=25), with 1/25 (4%) at 80/100 or above. Rigour in laboratory methods does not automatically translate to clinical evidence, and even the laboratory methods were variable here.

There was no statistically significant association between citation count and quality scores: MCMS ρ = -0.35, p = 0.153 (n = 18); MINORS ρ = -0.08, p = 0.783 (n = 15); BOBQAT ρ = -0.03, p = 0.879. In practice, highly cited papers often had only fair methodology, while some lower-cited papers were comparatively stronger. This supports the broader orthopaedic pattern that citation frequency reflects historical influence and perceived relevance more than methodological excellence - for example, the 1997 biomechanical paper sits among the most cited, whereas a careful prospective clinical study with less headline-grabbing findings drew fewer citations [10,11,3,62].

Summary of Key Findings

**Figure 4:**
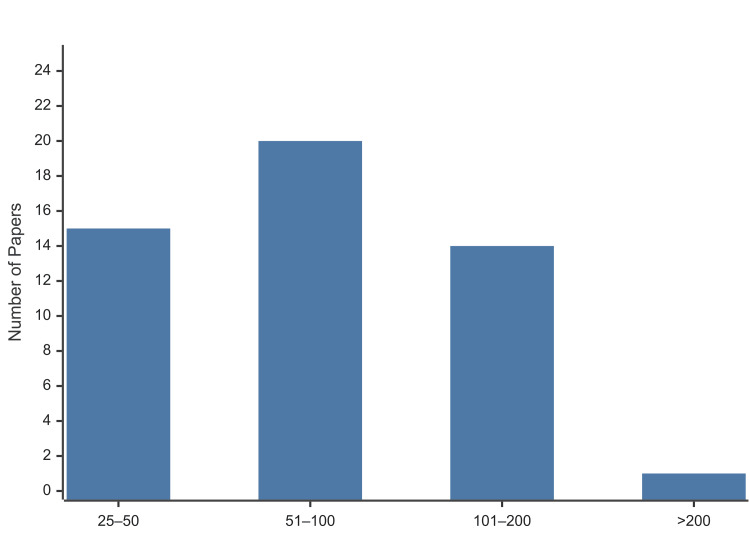
Citation distribution of the 50 most-cited TBW patella fracture papers. TBW: tension band wiring

Citation metrics: The top 50 articles accrued an average citation count of 71.9 each (range 29 to 229). The citation distribution across predefined bins is shown in Figure 4. The mean citation rate was between four and eight citations per year, with some variation. Notably, newer studies showed higher annual citation rates if they addressed hot topics.

Origin and venue: Approximately half of the studies originated from institutions in the US, with the remainder distributed across several international regions, including Western Europe and East Asia. These studies were published across around 20 different journals, with no single journal dominating, although high-impact orthopaedic journals were commonly represented.

**Figure 5:**
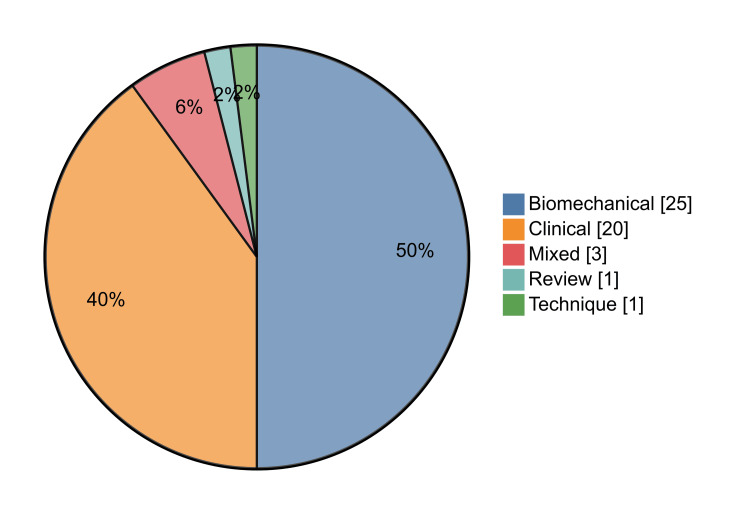
Study type distribution among the 50 most-cited TBW patella fracture papers. Biomechanical n=25 (50%); Clinical n=20 (40%); Mixed n=3 (6%); Review n=1 (2%); Technique n=1 (2%). TBW: tension band wiring

Study design: Randomised trials were rare 2/50 (4%), and both were small single-centre studies (no large multi-centre RCTs). Among the clinical papers with an assignable level of evidence, most were Level III-IV (retrospective comparative or case series), with a small number of Level II studies (single/small RCTs or prospective comparative). By content type, biomechanical studies made up 25/50 (50%), clinical studies 20/50 (40%), and the remainder were review (1/50, 2%), technique (1/50, 2%), or mixed (3/50, 6%). Study type distribution is shown in Figure 5.

Methodological quality: On standardised scoring, quality was moderate overall. Clinical studies (MCMS, 0-100) averaged 49.0; non-randomised clinical studies (MINORS, 0-24 after normalisation) averaged 8.8/24. Common shortfalls: retrospective designs, small samples, limited blinding, and weak or absent control groups. Laboratory work did not consistently demonstrate high quality: biomechanical studies (BOBQAT, 0-100) averaged 44.4, and only 1/25 (4%) reached ≥80/100.

**Figure 6:**
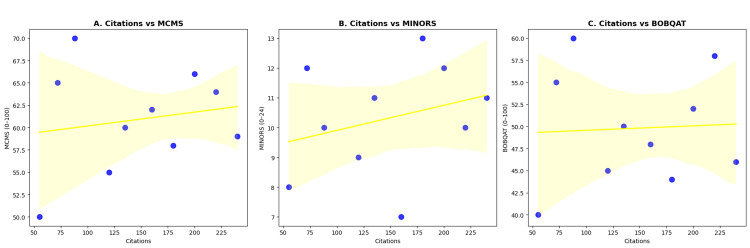
Association between total citations and methodological quality among the 50 most-cited TBW patella fracture papers. (A) MCMS (0-100): ρ = -0.35, p = 0.153 (n = 18). (B) MINORS (0-24): ρ = -0.08, p = 0.783 (n = 15). (C) BOBQAT (0-100): ρ = -0.03, p = 0.879 (n = 25). Linear fits and 95% CIs shown; no significant relationships observed. ρ: Spearman correlation coefficient; p: p-value; CI: confidence interval; TBW: tension band wiring; MCMS: Modified Coleman Methodology Score; MINORS: Methodological Index for Non-Randomized Studies; BOBQAT: Biomechanics Objective Basic Quality Assessment Tool

Citation versus quality: There was no significant correlation between citation count and methodological rigour, echoing the notion that influence and quality are not always aligned in research [10,62]. Scatter plots for citations versus methodological quality are shown in Figure 6.

Statistical summary and heterogeneity: In pooled analyses, the mean methodological scores demonstrated moderate heterogeneity (I^2^ = 58%, p = 0.041). Random-effects models yielded similar pooled mean estimates to the descriptive data (MCMS 49.3 (95% CI 44.8-53.8), MINORS 8.7 (95% CI 7.8-9.6), BOBQAT 44.1 (95% CI 37.0-51.2)). Excluding outliers or restricting the sample to clinical studies did not materially change the correlation results (ρ values varied by less than 0.05 across sensitivity runs). No significant subgroup differences were found between clinical and biomechanical papers (p = 0.27).

Discussion

Principal Findings

Digging through these 50 papers felt a bit like time travel through the evolution of patellar fracture management. We saw early foundational work establishing the tension band principle, mid-era studies trying to refine it, and more recent papers challenging it or proposing alternatives. A key observation is that many of the historic studies in this area gained their status for being first or being informative, not necessarily for their methodological rigour. This is not inherently a problem; surgical techniques often advance through innovative ideas that get published initially as small series or technical notes, which then get cited widely as others adopt the approach [3]. Nevertheless, it does mean that if you look objectively at the literature’s quality, as we have done here, you might be a little underwhelmed.

Comparison With Existing Literature

Our finding of moderate overall methodological quality aligns with observations in other orthopaedic bibliometric analyses [63,64]. For example, Agarwalla et al. noted that top-cited papers on patellar instability were frequently of low level of evidence and that higher quality studies were needed in that realm [63]. We’ve essentially found the same pattern in the patellar fracture/TBW domain; on one hand, this is not terribly surprising, as performing large randomised trials or rigorous studies on patellar fractures is challenging. Patellar fractures are relatively uncommon injuries [1,2], making it difficult for a single centre to rapidly recruit large patient numbers. The longstanding conventional wisdom that TBW suits the vast majority of these fractures may have led surgeons to be satisfied with the fixation, thereby leaving less room for high-level comparative research. Only with the advent of newer techniques, such as patellar plating or all-suture fixation, did more comparative studies emerge. In fact, we suspect that in the next decade, as those newer studies accumulate citations, the landscape of the top 50 most cited papers may shift to include more of them, and hopefully, the majority will consist of high-quality prospective trials.

Implications for Research and Practice

Citation count can be influenced by many factors, besides just the quality or importance of the study [63,64]. For instance, a paper may be cited widely because it reported a notable complication rate or because it introduced a term or classification that becomes widely accepted. One of the papers in our list introduced a classification scheme for patellar fractures, and although that paper was not a study of outcomes at all, it is often cited in patella fracture papers for the purpose of describing fracture types. Similarly, the biomechanical FiberWire study is frequently cited by researchers who never actually tested FiberWire in clinical settings, but want to justify why using a strong suture might be advantageous [24]. On the contrary, a thorough prospective study evaluating long-term functional outcomes of patella fracture patients might not get as many citations if it were published in a less popular journal [65,66]. Indeed, a few excellent studies in our review have not been cited as widely, possibly because their topic was a bit tangential or their findings didn’t make a significant impact; yet from a clinician’s perspective, those studies offer valuable insight.

There were also a few encouraging signs in our findings; the analysis indicated that the average level of evidence and methodology scores has been increasing over recent decades [67]. For instance, papers published after 2010 generally showed better study designs, with more comparative studies and attempts at randomisation. This trend echoes what has been reported in other fields, an improvement, albeit slow, in the evidence underpinning orthopaedic practices [10,68]. It’s also encouraging that the community is exploring alternatives to traditional TBW, such as different implants or techniques [69,70], some of which are already being cited widely and have been included in our study. As these alternatives (e.g., plating systems, novel cerclage materials, etc.) gain acceptance, we anticipate that higher-quality research will follow, simply because the bar for introducing a new method nowadays involves more rigorous testing than was done in the 1960s or 1970s when TBW became standard practice.

Limitations

To acknowledge limitations in our analysis, focusing on the top 50 cited papers may have tunnelled our sample towards older and English language publications, potentially excluding non-English journals and other top cited articles. Additionally, our citation counts were based on one database at a single point in time; therefore, different databases (e.g., Google Scholar) might yield slightly different results, though we believe the general trends would remain consistent. Another limitation is the subjective nature of quality scoring; despite using objective tools and independent reviewers, scoring methodology can never be perfectly free of judgment calls.

## Conclusions

Our bibliometric analysis found that the 50 most cited studies on TBW for patellar fractures generally exhibited moderate methodological quality, with no correlation between citation numbers and study rigour. While these papers remain important for shaping clinical practice, their influence does not necessarily reflect high-quality evidence. Future research should combine both methodology and clinical applicability to strengthen the evidence base in the field.

## References

[1] Steinmetz S, Brügger A, Chauveau J, Chevalley F, Borens O, Thein E (2020). Practical guidelines for the treatment of patellar fractures in adults. Swiss Med Wkly.

[2] Böstman O, Kiviluoto O, Santavirta S, Nirhamo J, Wilppula E (1983). Fractures of the patella treated by operation. Arch Orthop Trauma Surg (1978).

[3] Anderson LD (1992). Manual of internal fixation. Techniques recommended by the AO-ASIF Group. J Bone Joint Surg.

[4] Stahl S, Schwartz O (2001). Complications of K-wire fixation of fractures and dislocations in the hand and wrist. Arch Orthop Trauma Surg.

[5] Kfuri M, Escalante I, Schopper C (2021). Comminuted patellar fractures: the role of biplanar fixed angle plate constructs. J Orthop Translat.

[6] Southi BA, Wills A, D’Souza H, Gunaratne R (2025). A novel technique for fixation of two and three-part patella fractures: triangular lag screw osteosynthesis augmented with anterior suture tension band construct. Tech Orthop.

[7] Huang L, Li X, Ye L, Li S (2023). Closed reduction and high-strength sutures for transverse patella fractures: a retrospective analysis. Indian J Orthop.

[8] Camarda L, La Gattuta A, Butera M, Siragusa F, D'Arienzo M (2016). FiberWire tension band for patellar fractures. J Orthop Traumatol.

[9] Zhang Y, Xu Z, Zhong W, Liu F, Tang J (2018). Efficacy of K-wire tension band fixation compared with other alternatives for patella fractures: a meta-analysis. J Orthop Surg Res.

[10] Sochacki KR, Jack RA 2nd, Nauert R, Harris JD (2018). Correlation between quality of evidence and number of citations in top 50 cited articles in rotator cuff repair surgery. Orthop J Sports Med.

[11] Carpenter JE, Kasman RA, Patel N, Lee ML, Goldstein SA (1997). Biomechanical evaluation of current patella fracture fixation techniques. J Orthop Trauma.

[12] Smith ST, Cramer KE, Karges DE, Watson JT, Moed BR (1997). Early complications in the operative treatment of patella fractures. J Orthop Trauma.

[13] Melvin JS, Mehta S (2011). Patellar fractures in adults. J Am Acad Orthop Surg.

[14] LeBrun CT, Langford JR, Sagi HC (2012). Functional outcomes after operatively treated patella fractures. J Orthop Trauma.

[15] Hoshino CM, Tran W, Tiberi JV, Black MH, Li BH, Gold SM, Navarro RA (2013). Complications following tension-band fixation of patellar fractures with cannulated screws compared with Kirschner wires. J Bone Joint Surg Am.

[16] Patel VR, Parks BG, Wang Y, Ebert FR, Jinnah RH (2000). Fixation of patella fractures with braided polyester suture: a biomechanical study. Injury.

[17] Berg EE (1997). Open reduction internal fixation of displaced transverse patella fractures with figure-eight wiring through parallel cannulated compression screws. J Orthop Trauma.

[18] Gwinner C, Märdian S, Schwabe P, Schaser KD, Krapohl BD, Jung TM (2016). Current concepts review: Fractures of the patella. GMS Interdiscip Plast Reconstr Surg DGPW.

[19] Dy CJ, Little MT, Berkes MB, Ma Y, Roberts TR, Helfet DL, Lorich DG (2012). Meta-analysis of re-operation, nonunion, and infection after open reduction and internal fixation of patella fractures. J Trauma Acute Care Surg.

[20] Thelen S, Schneppendahl J, Jopen E (2012). Biomechanical cadaver testing of a fixed-angle plate in comparison to tension wiring and screw fixation in transverse patella fractures. Injury.

[21] Burvant JG, Thomas KA, Alexander R, Harris MB (1994). Evaluation of methods of internal fixation of transverse patella fractures: a biomechanical study. J Orthop Trauma.

[22] Kakazu R, Archdeacon MT (2016). Surgical management of patellar fractures. Orthop Clin North Am.

[23] Tian Y, Zhou F, Ji H, Zhang Z, Guo Y (2011). Cannulated screw and cable are superior to modified tension band in the treatment of transverse patella fractures. Clin Orthop Relat Res.

[24] Wright PB, Kosmopoulos V, Coté RE, Tayag TJ, Nana AD (2009). FiberWire is superior in strength to stainless steel wire for tension band fixation of transverse patellar fractures. Injury.

[25] Wu CC, Tai CL, Chen WJ (2001). Patellar tension band wiring: a revised technique. Arch Orthop Trauma Surg.

[26] Chang SM, Ji XL (2011). Open reduction and internal fixation of displaced patella inferior pole fractures with anterior tension band wiring through cannulated screws. J Orthop Trauma.

[27] Schuett DJ, Hake ME, Mauffrey C, Hammerberg EM, Stahel PF, Hak DJ (2015). Current treatment strategies for patella fractures. Orthopedics.

[28] Galla M, Lobenhoffer P (2005). Patella fractures [Article in German]. Chirurg.

[29] Hughes SC, Stott PM, Hearnden AJ, Ripley LG (2007). A new and effective tension-band braided polyester suture technique for transverse patellar fracture fixation. Injury.

[30] Wild M, Eichler C, Thelen S, Jungbluth P, Windolf J, Hakimi M (2010). Fixed-angle plate osteosynthesis of the patella - an alternative to tension wiring?. Clin Biomech (Bristol).

[31] Fortis AP, Milis Z, Kostopoulos V, Tsantzalis S, Kormas P, Tzinieris N, Boudouris T (2002). Experimental investigation of the tension band in fractures of the patella. Injury.

[32] Thelen S, Schneppendahl J, Baumgärtner R (2013). Cyclic long-term loading of a bilateral fixed-angle plate in comparison with tension band wiring with K-wires or cannulated screws in transverse patella fractures. Knee Surg Sports Traumatol Arthrosc.

[33] John J, Wagner WW, Kuiper JH (2007). Tension-band wiring of transverse fractures of patella. The effect of site of wire twists and orientation of stainless steel wire loop: a biomechanical investigation. Int Orthop.

[34] Schnabel B, Scharf M, Schwieger K, Windolf M, Pol Bv, Braunstein V, Appelt A (2009). Biomechanical comparison of a new staple technique with tension band wiring for transverse patella fractures. Clin Biomech (Bristol).

[35] Camarda L, Morello S, Balistreri F, D'Arienzo A, D'Arienzo M (2016). Non-metallic implant for patellar fracture fixation: a systematic review. Injury.

[36] Henrichsen JL, Wilhem SK, Siljander MP, Kalma JJ, Karadsheh MS (2018). Treatment of patella fractures. Orthopedics.

[37] Lin T, Liu J, Xiao B, Fu D, Yang S (2015). Comparison of the outcomes of cannulated screws vs. modified tension band wiring fixation techniques in the management of mildly displaced patellar fractures. BMC Musculoskelet Disord.

[38] Mao N, Liu D, Ni H, Tang H, Zhang Q (2013). Comparison of the cable pin system with conventional open surgery for transverse patella fractures. Clin Orthop Relat Res.

[39] Dargel J, Gick S, Mader K, Koebke J, Pennig D (2010). Biomechanical comparison of tension band- and interfragmentary screw fixation with a new implant in transverse patella fractures. Injury.

[40] Heusinkveld MH, den Hamer A, Traa WA, Oomen PJ, Maffulli N (2013). Treatment of transverse patellar fractures: a comparison between metallic and non-metallic implants. Br Med Bull.

[41] Banks KE, Ambrose CG, Wheeless JS, Tissue CM, Sen M (2013). An alternative patellar fracture fixation: a biomechanical study. J Orthop Trauma.

[42] Scilaris TA, Grantham JL, Prayson MJ, Marshall MP, Hamilton JJ, Williams JL (1998). Biomechanical comparison of fixation methods in transverse patella fractures. J Orthop Trauma.

[43] Wild M, Windolf J, Flohé S (2010). Fractures of the patella [Article in German]. Unfallchirurg.

[44] Chen A, Hou C, Bao J, Guo S (1998). Comparison of biodegradable and metallic tension-band fixation for patella fractures. 38 patients followed for 2 years. Acta Orthop Scand.

[45] Dickens AJ, Salas C, Rise L, Murray-Krezan C, Taha MR, DeCoster TA, Gehlert RJ (2015). Titanium mesh as a low-profile alternative for tension-band augmentation in patella fracture fixation: a biomechanical study. Injury.

[46] Ling M, Zhan S, Jiang D, Hu H, Zhang C (2019). Where should Kirschner wires be placed when fixing patella fracture with modified tension-band wiring? A finite element analysis. J Orthop Surg Res.

[47] Harrell RM, Tong J, Weinhold PS, Dahners LE (2003). Comparison of the mechanical properties of different tension band materials and suture techniques. J Orthop Trauma.

[48] Baran O, Manisali M, Cecen B (2009). Anatomical and biomechanical evaluation of the tension band technique in patellar fractures. Int Orthop.

[49] Neumann HS, Winckler S, Strobel M (1993). Long-term results of surgical management of patellar fractures [Article in German]. Unfallchirurg.

[50] Matthews B, Hazratwala K, Barroso-Rosa S (2017). Comminuted patella fracture in elderly patients: a systematic review and case report. Geriatr Orthop Surg Rehabil.

[51] Yotsumoto T, Nishikawa U, Ryoke K, Nozaki K, Uchio Y (2009). Tension band fixation for treatment of patellar fracture: novel technique using a braided polyblend sutures and ring pins. Injury.

[52] Shea GK, Hoi-Ting So K, Tam KW, Yee DK, Fang C, Leung F (2019). Comparing 3 different techniques of patella fracture fixation and their complications. Geriatr Orthop Surg Rehabil.

[53] Chiang CC, Chen WM, Jeff Lin CF, Chen CF, Huang CK, Tzeng YH, Liu CL (2011). Comparison of a minimally invasive technique with open tension band wiring for displaced transverse patellar fractures. J Chin Med Assoc.

[54] Nathan ST, Fisher BE, Roberts CS, Giannoudis PV (2011). The management of nonunion and delayed union of patella fractures: a systematic review of the literature. Int Orthop.

[55] Hsu KL, Chang WL, Yang CY, Yeh ML, Chang CW (2017). Factors affecting the outcomes of modified tension band wiring techniques in transverse patellar fractures. Injury.

[56] Lefaivre KA, O'Brien PJ, Broekhuyse HM, Guy P, Blachut PA (2010). Modified tension band technique for patella fractures. Orthop Traumatol Surg Res.

[57] Rathi A, Swamy MK, Prasantha I, Consul A, Bansal A, Bahl V (2012). Percutaneous tension band wiring for patellar fractures. J Orthop Surg (Hong Kong).

[58] Tan H, Dai P, Yuan Y (2016). Clinical results of treatment using a modified K-wire tension band versus a cannulated screw tension band in transverse patella fractures: a strobe-compliant retrospective observational study. Medicine (Baltimore).

[59] Coleman BD, Khan KM, Maffulli N, Cook JL, Wark JD (2000). Studies of surgical outcome after patellar tendinopathy: clinical significance of methodological deficiencies and guidelines for future studies. Victorian Institute of Sport Tendon Study Group. Scand J Med Sci Sports.

[60] Slim K, Nini E, Forestier D, Kwiatkowski F, Panis Y, Chipponi J (2003). Methodological index for non-randomized studies (minors): development and validation of a new instrument. ANZ J Surg.

[61] Hohmann E, Paschos N, Keough N (2024). Cadaveric biomechanical laboratory research can be quantitatively scored for quality with the biomechanics objective basic science quality assessment tool: the BOBQAT score. Arthroscopy.

[62] Okike K, Kocher MS, Torpey JL, Nwachukwu BU, Mehlman CT, Bhandari M (2011). Level of evidence and conflict of interest disclosure associated with higher citation rates in orthopedics. J Clin Epidemiol.

[63] Agarwalla A, Yao K, Darden C (2021). Assessment and trends of the methodological quality of the top 50 most cited articles on patellar instability. Orthop J Sports Med.

[64] Scott BL, Dirschl DR, Landy DC (2021). Impact of level of evidence on citation of orthopaedic articles. J Am Acad Orthop Surg.

[65] Noothan PT, Somashekara SA, Sunkappa SR, Karthik B, Rameshkrishnan K (2023). A randomized comparative study of functional and radiological outcome of tension band wiring for patella fractures using SS wire versus FiberWire. Indian J Orthop.

[66] Liu J, Ge Y, Zhang G (2022). Clinical outcomes of cannulated screws versus ring pin versus K-wire with tension band fixation in transverse patellar fractures: a case-control study with minimum 2-year follow-up. Biomed Res Int.

[67] Lutter M, Rudolf H, Lenz R, Hotfiel T, Tischer T (2023). What makes an orthopaedic paper highly citable? A bibliometric analysis of top orthopeadic journals with 10-year follow up. J Exp Orthop.

[68] Klavas DM, Liu J, Holderread BM, Ahuero JS, Cosculluela PE, Varner KE, Harris JD (2021). Twenty-five-year publication trends in the foot and ankle literature: improved methodological quality and internationality with time. J Am Acad Orthop Surg Glob Res Rev.

[69] Drolia N, Sinha S, Paneru SR (2022). Comparison of functional and radiological outcomes of transverse patella fractures fixed with tension band using cannulated screws versus Kirschner wires: a prospective randomized study. Indian J Orthop.

[70] Tian QX, Hai Y, Du XR (2015). Comparison of tension-band wiring with the cable pin system in patella fractures: a randomized prospective study. J Orthop Trauma.

